# Oxidative State in Cutaneous Melanoma Progression: A Question of Balance

**DOI:** 10.3390/antiox13091058

**Published:** 2024-08-30

**Authors:** Mascia Benedusi, Heaji Lee, Yunsook Lim, Giuseppe Valacchi

**Affiliations:** 1 Department of Neuroscience and Rehabilitation, University of Ferrara, 44121 Ferrara, Italy; mascia.benedusi@unife.it; 2 Department of Food and Nutrition, Kyung Hee University, Seoul 02447, Republic of Korea; ji3743@khu.ac.kr (H.L.); ylim@khu.ac.kr (Y.L.); 3 Plants for Human Health Institute, NC Research Campus, NC State University, Kannapolis, NC 28081, USA; 4 Department of Environmental and Prevention Sciences, University of Ferrara, 44121 Ferrara, Italy

**Keywords:** reactive oxygen species (ROS), human cancers, melanoma progression, antioxidant/pro-oxidant therapies

## Abstract

Reactive oxygen species (ROS) are highly bioactive molecules involved not only in tissue physiology but also in the development of different human conditions, including premature aging, cardiovascular pathologies, neurological and neurodegenerative disorders, inflammatory diseases, and cancer. Among the different human tumors, cutaneous melanoma, the most aggressive and lethal form of skin cancer, is undoubtedly one of the most well-known “ROS-driven tumor”, of which one of the main causes is represented by ultraviolet (UV) rays’ exposure. Although the role of excessive ROS production in melanoma development in pro-tumorigenic cell fate is now well established, little is known about its contribution to the progression of the melanoma metastatic process. Increasing evidence suggests a dual role of ROS in melanoma progression: excessive ROS production may enhance cellular growth and promote therapeutic resistance, but at the same time, it can also have cytotoxic effects on cancer cells, inducing their apoptosis. In this context, the aim of the present work was to focus on the relationship between cell redox state and the signaling pathways directly involved in the metastatic processes. In addition, oxidative or antioxidant therapeutic strategies for metastatic melanoma were also reviewed and discussed.

## 1. Introduction

Reactive oxygen species (ROS) are continuously generated in eukaryotic cells by cellular metabolism [1,2]. Prominent ROS include hydroxyl radical (HO·) and superoxide anion radical (O_2_^•−^), hydrogen peroxide (H_2_O_2_), and singlet oxygen (^1^O_2_) [3]. It has long been known that these molecules, because of their high reactivity, can damage important cellular structures, including proteins, lipids, and nucleic acids, often resulting in permanent functional alterations [3]. At low to moderate levels, ROS play important roles in several physiological processes, including modulation of cell survival, differentiation, cell signaling, fighting pathogens, modulating wound healing, and inflammation. However, elevated ROS has been implicated in the development or in the exacerbation of many human diseases, including cancer. Accumulating evidence suggests that cancer cells are characterized by aberrant redox homeostasis, but the role of this pro-oxidative state is still controversial since, during different stages of tumor progression, altered ROS levels play contradictory roles in both cell growth and apoptosis [4].

Indeed, high ROS levels seem to contribute to early events that involve cancer initiation and progression, inducing the proliferation of cancer cells. At the same time, this high ROS level appears to be cytotoxic during the late phases of tumor progression and metastasis, suggesting a possible therapeutical approach for human cancer treatment [5].

Starting from this well-recognized knowledge, this review intends to highlight the role of ROS in the development and progression of a very aggressive type of human cancer, cutaneous melanoma, which is defined as a “ROS-driven” human tumor [6]. Specifically, it discusses the relationship between oxidative stress and different stages of melanoma development and progression from both the molecular and clinical point of view with the aim of clarifying the apparently anomalous role of antioxidants in preventing and treating this malignant condition.

## 2. Redox Mediators and Sources

ROS and reactive nitrogen species (RNS) are the two free radicals that originate from both exogenous and endogenous sources that play an essential role in the maintenance of homeostasis in biological systems. ROS in living organisms is produced mainly from the electron transport chain of mitochondria, peroxisomes, endoplasmic reticulum, and plasma membranes [7]. In addition, ROS are enzymatically produced by oxidases, including nicotinamide adenine dinucleotide phosphate (NADP)H oxidase (NOX), lipid oxidase, cyclooxygenases (COXs), and xanthine oxidase. 

NOX family is composed of membrane-bound proteins that act by trans-porting electrons through biological membranes to reduce oxygen of O_2_^•−^ to H_2_O_2_. Specifically, these enzymes catalyze a biological reaction by transferring the electron from NADPH to O_2_, resulting in the generation of ROS, including O_2_^•−^, H_2_O_2_, and HO· [8].

### 2.1. ROS Produced by Cellular Organelles

As clearly described by Agustin and Enríquez [9], ROS can be defined as the product of subsequent one-electron reduction of oxygen. Indeed, by subsequent steps, first, O_2_^•−^ is produced, and then it is converted into H_2_O_2_, which, in turn, is converted into the highly harmful ROS HO·, which governs different toxic reactions, such as the Fenton reaction. 

The pioneering works by Chance and colleagues argued that mitochondria produce more than 90% of cellular ROS through the electron transport chain, which generates a large amount of O_2_^•−^, as extensively reviewed elsewhere [10,11,12,13].

Interestingly, over the last two decades, this concept has been questioned, and it has been proposed that quantitatively, endoplasmic reticulum (ER) and peroxisomes have a greater ability to produce ROS than mitochondria themselves [14]. 

ER is a multi-functional organelle involved in calcium storage, protein synthesis, transport and folding, lipid synthesis, and carbohydrate metabolism [15]. Deregulated calcium signaling in ER leads to mitochondrial dysfunction and NOX hyperactivity, which, in turn, drives the augmented cellular ROS level. Within the family of NOX, the constitutively active NOX4 is predominantly localized in the endoplasmic reticulum, which produces H_2_O_2_ and contributes to the vicious cycle of oxidative stress in this organelle. Ultimately, the disruption of redox homeostasis in the ER is involved in the pathogenesis of various human diseases, such as neurodegenerative diseases (Alzheimer’s disease, Parkinson’s disease), Type 1 and Type 2 diabetes, atherosclerosis, and liver diseases, among many others [16,17].

Another important source of endogenous ROS is the peroxisomes; specifically, the production of ROS in these organelles involves dynamic and metabolic pathways, including fatty acid oxidation, photorespiration, purine catabolism, and isoprenoid biosynthesis [3]. 

### 2.2. Exogenous Sources of ROS

Some of the exogenous sources of ROS involve nutrients, drugs, physical stress (UV light, X-rays, and gamma rays), air pollution, and metals (Fe, Cu, Co, and Cr) [2,18,19,20]. Radiations and chemotherapeutic agents can evoke oxidative damage to proteins, lipids, and deoxyribonucleic acid (DNA) and then increase ROS, causing damage to blood vessels and hematopoietic systems. In addition, they are known not only to increase lipid peroxidation but also to suppress the levels of antioxidant enzymes involved in ROS detoxification [21].

### 2.3. Endogenous Sources of RNS

RNS includes nitric oxide (NO^•^), nitrogen dioxide (^•^NO_2_), and peroxynitrite (ONOO^−^). Nitric oxide is produced from L-arginine by three main isoforms of nitric oxide synthase (NOS): epithelial NOS (eNOS), neuronal NOS (nNOS), and inducible NOS (iNOS). While the production of NO by eNOS and nNOS is strictly regulated by calcium level through a calmodulin-dependent mechanism, iNOS is induced in response to infection and/or inflammation and is not modulated by calcium [22]. 

### 2.4. Exogenous Sources of RNS

NO_2_ is a primary pollutant in both indoor and outdoor air produced by the combustion of fossil fuels. Emissions from cars and fossil fuels are the major sources of ·NO_2_ in outdoor air. Regarding the ·NO_2_ found in indoor air, it derives from gas stoves, kerosene heaters, building heating, and tobacco smoke [23]. 

Both ROS and RNS at low cellular concentrations are considered regulatory mediators in signaling processes, while at high concentrations, they have harmful effects on human health, altering fundamental cellular molecules, such as proteins and lipids and promoting the development of different human diseases, including cancer.

## 3. Role of ROS in Physiological and Pathological Processes: A Matter of Balance between ROS Production and Efficiency of Antioxidant Cellular System

ROS are known to have an essential role in many metabolic pathways. Indeed, ROS participates in many redox regulatory cell activities to maintain cellular homeostasis as promoters of natural defenses [24]. Particularly, ROS regulates inflammatory signaling, apoptotic/autophagic pathways, fibrosis, cell proliferation, cell survival, and a variety of other physiological processes. These processes are mediated by transcription factors, which can be directly regulated by ROS and, in turn, can interact with specific DNA motifs on promoters of target genes, modifying gene expression profiles and cellular responses to oxidative stress [25,26].

On the other hand, ROS overproduction disrupts the redox homeostasis, causing cellular and tissue damage [1]. Therefore, the balance between levels of ROS generation and levels of ROS detoxification is invaluable to prevent the disruption of normal cellular homeostasis, which causes oxidative damage to cells. 

Accordingly, cells have evolved an antioxidant system to neutralize excess ROS production [1]. It can be categorized by enzymatic antioxidants like superoxide dismutase (SOD), catalase (CAT) and glutathione peroxidase (GPx), NADPH-quinone oxidoreductase-1 (NQO1), and heme-oxygenase (HO-1) and non-enzymatic antioxidants, such as vitamins (vitamins A, C, E, and K), minerals (Zn, Fe, Cu, and Se), nitrogen compounds (uric acid), and glutathione (GSH), as well as exogenous diets, such as polyphenols (phenolic acid and flavonoids) [27]. The cellular antioxidant system is not infallible; in fact, while under physiological conditions, it could efficiently counteract the potential negative effect of ROS, but when ROS production reaches an excessive level, the pro-oxidant and antioxidant imbalance results in oxidative damage [28], including DNA, oxidation, lipid peroxidation of polyunsaturated fatty acids (PUFAs), and the oxidation of proteins. As a result, cellular components’ functions might be impaired.

## 4. Redox Sensitive Pathways and Cancer Progression

Concerning the role of ROS and relative oxidative stress status in cancer, they have long been considered mutagens involved in tumor development. 

Nevertheless, nowadays, the function of oxidative stress in cancer progression is still controversial [29]. 

### 4.1. Involvement of ROS in Cancer Cells Proliferation

The high proliferation rate of cancer cells is associated with high ROS production that overcomes endogenous antioxidant response, promoting different signaling pathways associated with enhanced cell proliferation, such as the phosphoinositide 3-kinase(PI3K)/Akt and mitogen-activated protein kinase (MAPK) [30]. ROS can trigger the PI3K/Akt signaling cascade by inactivating the tensin homolog (PTEN) [31], which negatively controls this pathway. In a vicious cycle, the activation of Akt induces an increase of cellular ROS to further promote tumoral cell survival and proliferation [32]. A growing set of data provides evidence that ROS, particularly H_2_O_2_, acts as signaling molecules within cancer cells [33]. The direct oxidation of target proteins by H_2_O_2_ seems quite improbable in the presence of reactive antioxidants. Lee [34] demonstrated that H_2_O_2_ can induce a reversible inactivation of PTEN since it is rapidly reduced back to its active form by the antioxidant thioredoxin (Trx) system.

**Figure 1:**
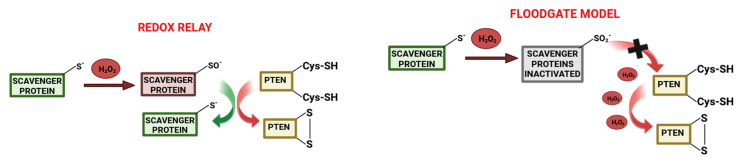
Possible mechanisms for H_2_O_2_-dependent regulation of PTEN. Left panel: the redox relay mechanism uses scavenging enzymes, such as the thiol peroxidases peroxiredoxins or glutathione peroxidases, characterized by a high intrinsic H_2_O_2_ thiol reactivity to transduce the H_2_O_2_ signal and oxidize PTEN. Indeed, these enzymes function not just to eliminate H_2_O_2_ but also act as sensors. They can transfer oxidizing equivalents to less reactive thiol proteins. Right panel: in the floodgate model, H_2_O_2_ inactivates the abovementioned scavengers (viz., through hyper-oxidation to sulfinic (SO^2−^) acid) to allow for H_2_O_2_-mediated oxidation of PTEN. Created with Biorender.com accessed on 1 July 2024.

H_2_O_2_ can oxidize target proteins, such as PTEN [35], through two alternative mechanisms: the “redox relay” or the “floodgate model”. In the first case [36], through a two-step process, the scavenging enzymes (viz., peroxiredoxins, which are characterized by high H_2_O_2_ reactivity), after being in contact with H_2_O_2_, transfer the oxidation to the target proteins through the formation of a mixed disulfide bond between the scavenging enzymes and the interacting protein itself; interestingly, in this proposed model, the reduction in peroxiredoxins causes a decrease in the H_2_O_2_-induced oxidation of redox-regulated proteins instead of inducing an increase. In the floodgate model [37], the hyper-oxidation of peroxiredoxins, due to rapid and localized increases in H_2_O_2_, decreases the peroxidase activity, inducing an accumulation of H_2_O_2_ available for oxidation of less sensitive proteins within this redox microenvironment (Figure 1). 

Furthermore, ROS may enhance cancer cells’ survival through the activation of nuclear factor kappa-light-chain-enhancer of activated B cells (NF-κB). The constitutive activation of NF-κB is frequently observed in different human cancer cells, making this a hallmark of cancer [38]. There is increasing evidence in the literature that confirms the association between high ROS levels, NF-κB activation, and cancer progression. It has been recently demonstrated that in gastric cancer cell lines, melatonin, by reducing the production of cellular ROS, induced a decrease in p-p65 expression level and of NF-κB target proteins Matrix Metalloproteinases 2 (MMP2) and cyclin D1, which are directly involved in cancer proliferation and metastasis [39]. In addition, in a different study, it was observed that CR6-interacting factor 1 (Crif1), a novel factor involved in the assembly of oxidative phosphorylation (OXPHOS) complexes in mitochondria, promotes the hepatocellular carcinoma growth by the redox activation of NF-κB pathway [40], confirming, therefore, the role of NF-κB in cancer progression.

Recent studies have shown that ROS can promote cells proliferation also by controlling microRNA (miRNAs). miRNAs are small (approximately 22 nucleotides) non-coding RNA molecules that regulate gene expression post-transcriptionally [41]. Convincing evidence has confirmed that miRNAs are dysregulated in tumors, making them possible biomarkers for human cancer diagnosis and prognosis [42]. Altered levels of miRNA have been detected in different human fluids, such as urine, blood, bronchial lavage, synovial fluid, milk, saliva, and cerebrospinal fluid [43,44].

Based on their function on cancer behavior, they are classified as OncomiRs, which stimulate tumor cell proliferation and metastasis, and tumor-suppressor miRNAs, which downregulate cancer progression. Interestingly, numerous miRNAs have dual effects and can act as tumor suppressors in some cancers and as tumor activators in other cancers. Different genome-wide profiling studies performed on various human cancers have demonstrated that cancer cells presented distinct miRNA profiles compared with normal cells and that miRNA expression in tumor samples seems lower than in normal tissues [45]. Since, in many cases, miRNAs also show tissue-specific expression, their use as cancer biomarkers during cancer treatment seems to be clinically relevant [46]. In this context, ROS has been recognized as a driver of abnormal miRNA expression in cancer cells, and several ROS-related miRNAs involved in cancer progression have been described [47]. Intracellular ROS can inhibit or induce miRNA expression through different mechanisms. First, ROS can induce epigenetic alteration of miRNA, such as hypermethylation or histone modification. Specifically, the methylation of the miRNA promoter region controls its expression. 

DNA methylation, which has a crucial role in controlling gene expression, occurs in cytosine bases located in CpG dinucleotides (cytosine nucleotide followed by a guanine nucleotide), also known as CpG islands, which are located in the proximal promoter regions of many genes [48]. As described by Morales et al. [49], the frequency of human miRNA gene methylation is significantly higher than that of protein-encoding genes. The direct involvement of ROS and miRNA gene methylation was demonstrated in ovarian cancer cells. In this human cancer, ROS inhibits miR-199a and miR-125b expression, and the promoter regions of both miRNA genes are hypermethylated upon H_2_O_2_ exposure [50]. 

Regarding the post-translational modifications of histones, they mediate different biological processes through the expression or repression of target genes. The most well-known histone modifications are acetylation, methylation, and phosphorylation, but very recently, Zhao and Garcia summarized other possible modifications, such as ubiquitination, citrullination, deamination, formylation, and others [51]. These epigenetic alterations, eventually driven by ROS, may characterize not only the protein-coding DNA sequences but also miRNA genes in their non-coding regions. ROS can regulate the activity of the histone deacetylases (HDACs), enzymes that catalyze the deacetylation of lysine residues of DNA-binding histone proteins, resulting in the chromatin condensation and downmodulation of gene expression [52].

Ago and colleagues demonstrated that in the heart, the ROS-induced oxidation of specific cysteines in HDACs (Cys-667 and Cys-669) results in its nuclear export that can be inverted by antioxidant Trx overexpression [53]. In lung cancer, it has been suggested that an autoregulatory loop including Nrf2, HDAC4, miR-1, and miR-206 may play a key role in the progression of lung cancer. In this paper, the authors first demonstrated that in lung cancer, the inactivation of Nrf2 leads to the inhibition of tumor growth, while the gain of function of this transcription factor favors the cancer cells’ metabolic reprogramming associated with tumor progression (viz., increasing the pentose phosphate pathway (PPP) and the tricarboxylic acid (TCA) cycle). Moreover, the authors asserted that this effect is mediated by the Nrf2 regulation of miR-1 and miR-206, two miRNA species whose expression is downregulated in different human cancers, such as lung, breast, and prostate [54,55]. Specifically, the authors suggested a Nrf2-dependent epigenetic regulation of miR-1 and miR-206 through the inhibition of HDAC4; indeed, the authors found a decreased HDAC4 mRNA expression and an augmented expression level of miR-1 and miR-206 in Nrf2-deficient cells. Therefore, based on the abovementioned results of Ago and colleagues [53], they also suggested that, in this case, the excessive ROS levels in Nrf2-deficient lung cells probably induce the oxidation of HDCA4 cysteines residues, promoting its cytoplasmic translocation and the subsequent induction of miR-1 and miR-206 gene expression [56]. 

As mentioned, there are different ROS-regulated miRNAs involved in cancer development and progression [57], but nowadays, only a few studies have focalized on the epigenetic regulation of miRNA expression by ROS, making this aspect still open to further more deeply in studies. Finally, ROS can regulate miRNA through specific transcription factors (viz., p53, NF-κB, Forkhead box O (FOXO), hypoxia-inducible factor (HIF)) or can directly/indirectly regulate Drosha and Dicer, the two major enzymes involved in miRNA biogenesis [47].

The fundamental role of ROS/Kelch-like ECH-associated protein 1 (Keap1)/nuclear factor erythroid 2-related factor 2 (Nrf2)/miRNA34a/b/c axis in regulating the tumor suppressive effects of curcumin has been observed in colorectal cancer [58]. In ovarian cancer, ROS induces the overexpression of ERBB2 and ERBB3, two well-known receptors of tyrosine-protein kinase involved in human cancer progression [59] through miR-199a and miR-125b repression due to DNA hypermethylation [50]. In liver cancer, ROS-induced miRNA 121 causes an increase in tumor migration and invasion through a significant decrease in PTEN expression [60].

### 4.2. Involvement of ROS in Cancer Metastasis

Besides cancer proliferation, different aspects are involved in the cancer metastasis cascade. Indeed, metastasis is a dynamic process during which the tumoral cells increase their proliferative potential, stimulate angiogenesis, detach from the primary tumor, survive in the circulatory torrent, gain invasive potential, and finally, grow in distant organs. 

In this complex cascade, a key event is angiogenesis, which is primarily induced by the vascular endothelial growth factor (VEGF) and is regulated at the transcriptional level by HIF-1 in response to hypoxia. The high proliferation rate of cancer cells normally induces hypoxia of the cells located in the central part of the tumor, with a consequent release of pro-angiogenic molecules and neovascularization. The master regulator of this specific step of tumor progression is, indeed, HIF-1, which is constituted of two distinct parts: an oxygen-sensitive HIF-1α subunit and a constitutive HIF-1β subunit [61]. In normoxia, the prolyl hydroxylases (PHDs) hydroxyls HIF-1α, inducing its polyubiquitylation and consequent rapid proteosomal degradation. When oxygen tension decreases, the hydroxylase activity of PHDs decreases, and HIF-1α proteosomal degradation is prevented, allowing its nucleus translocation and finally binding to HIF-1β. Then, HIF-1 can promote the expression of specific target genes, such as VEGF, that are involved in the angiogenic process. It has been demonstrated that ROS contributes to stabilizing HIF-1α, inhibiting its proteolysis [62,63]. In ovarian cancer, the high ROS level, mainly represented by H_2_O_2_, due to elevated NOX4 expression, correlated with enhanced angiogenesis and tumor growth. A high ROS level is fundamental for HIF-1α upregulation, which in turn augments VEGF mRNA and protein expression and subsequent angiogenesis [64]. Recently, it has also been shown that, in A2780 ovarian cancer cells, in turn, HIF-1α promotes the production of ROS mediated by NOX4 via an alternative splicing mechanism, emphasizing how this positive feedback process is involved in cancer angiogenesis and tumor progression [65]. Although angiogenesis is required for cancer metastasis, the degradation of the extracellular matrix (ECM) and the epithelial to mesenchymal transition (EMT) are fundamental to increasing cellular migration rate and for intracellular adaptation to survive in the blood torrent and colonize distant organs. Indeed, both processes are associated with cellular and molecular modifications that result in loss of cell–cell adhesion that, in turn, induces migration and invasion of tumoral cells [66]. The ECM is composed of distinct components, including proteins, glycoproteins, proteoglycans, and GAGs; during development, ECM remodeling is finely regulated, but a deregulated change in its composition can affect its biochemical properties, inducing carcinogenesis and promoting the development of a tumoral microenvironment. A key role in this process is played by proteolytic enzymes belonging to three different families: the MMPs, the serine proteases and the cysteine proteases. It has been demonstrated that the proteolytic degradation of the ECM is promoted by H_2_O_2_ generated by the dismutation of NOX-derived superoxide, which, in turn, increases MMP expression levels and activity through the regulation of different intracellular redox-sensitive pathways [67]. Moreover, it has been shown that ROS also promotes the formation of functional invadopodia, specialized cell surface structures associated with degradation of the ECM during cancer invasiveness and metastasis [68]. 

During cancer progression, the deregulation of ECM contributes to EMT, a process that involves loss of epithelial cell–cell junction, which is associated with the decreased expression of epithelial markers like E-cadherin and the increased expression of mesenchymal markers (viz., fibronectin, vimentin, fibronectin, N-Cadherin) and MMPs (MMP-2, MMP-3, MMP-9) [69]. This process is mediated by the orchestrated activity of EMT-activating transcription factors, including TWIST1, TWIST 2, SNAIL, SLUG, ZEB1, and ZEB2. Moreover, these transcription factors are regulated by other cellular signaling pathways, such as NF-κB, TGF-β, and HIF-1 [70]. All these proteins can also be modulated by ROS, as described in the reviews by Cannito and colleagues [71] and by Farahzadi et al. [72]. 

Although primary tumors can discard millions of cells into the circulatory torrent, the colonization of distant organs is a rare event. The cancer population protagonist of the metastatic process is defined as metastatic cancer cells (MCCs). In an elegant paper, Cencioni and colleagues [73] described in detail how ROS can also influence the final success rate of the metastatic process. They elegantly explained how the journey of the MCCs into the blood is supported by epithelial cells, polymorphonuclear cells, and platelets and how these cells-producing ROS contribute to maintaining the metastable characteristics of MCCs. The authors also illustrated how the colonization of the new target organs starting from the “metastatic niches” is once again ROS-dependent. Indeed, the metastatic niche is formed by epithelial cells, fibroblasts, infiltrating leucocytes, and other tissue-specific cells that produce ROS inside the niche itself, regulating the metastatic cascade and processes [73].

## 5. Redox Sensitive Pathways Involved in Cancer Prevention

Besides the pro-tumorigenic role of ROS, several studies have reported that decreased oxidative stress is critical for metastasis; therefore, the function of ROS in cancer has a double-edged mechanism. Very excessive levels of ROS play an antiproliferative role in cancer cells since they can cause cycle arrest, senescence, and, finally, cell death [4]. Therefore, the antitumoral effects of ROS also need to be taken into consideration. As described by Wang et al. [29], ROS can promote different cell death mechanisms, including apoptosis, necroptosis, and ferroptosis [74]. As it concerns the apoptotic process, ROS drives both intrinsic and extrinsic pathways. The intrinsic pathway is triggered in a mitochondria-dependent manner by the release of several pro-apoptotic factors, including cytochrome-c (Cyt-c), from the mitochondrial intermembrane space into the cytosol. Once in the cytoplasm, these factors may activate the caspase cascade [75]. The phospholipid cardiolipin is involved in this process. Indeed, within the mitochondria, Cyt-c is bound to the mitochondrial inner membrane by its association with cardiolipin, but when cardiolipin is oxidized by ROS, its affinity for Cyt-c is attenuated, leading to Cyt-c release into the cytosol. Here, Cyt-c binds to and activates apoptotic protease activating factor 1 (Apaf-1) and procaspase-9, promoting the caspase 9 activation, which is the last step of the apoptotic process. Li and colleagues demonstrated that zinc oxide nanoparticles (ZnO NPs), an innovative antitumoral agent, significantly inhibit human multiple myeloma cell proliferation and enhance apoptosis, increasing ROS level, which, in turn, induces both mRNA and protein expression of Cyt-C, Apaf-1, caspase-9, and caspase-3. Moreover, while ZnO NPs disrupt mitochondrial function homeostasis in tumoral cells, they have low cytotoxic effects on peripheral blood mononuclear cells, suggesting their use in the treatment of human multiple myeloma [76]. In an interesting paper, based on the antitumoral effect of a light-induced ROS generator containing pyridinium (TBTP), it was shown how TBTP, accumulated in mitochondria, triggers a ROS burst, which is able to promote Cyt-c release and the activation of caspase-3/9, leading to cellular apoptosis [77].

On the other hand, the extrinsic apoptosis process involves specific cell membrane proteins known as death receptors (DRs), such as Fas, TNFR1, TNFR2, and the TRAIL receptors DR4 and DR5, which transfer the death signal from the extracellular ligands to the intracellular caspase machinery. This activation can be inhibited by the antiapoptotic factor cellular FLICE-inhibitory protein (c-FLIP). It has been proven that in prostate cells, ROS induces post-translational modification of c-FLIP, regulating its stability and increasing the sensitivity of cancer cells to the TNF family death ligand TRAIL [78]. More recently, it has been shown that in neck squamous cell carcinoma (AMC-HN4), thioridazine plus curcumin induce the downregulation of c-FLIP in a proteasome-dependent manner: increased proteasome activity due to the upregulation of proteasome subunit alpha 5 (PSMA5) expression via NOX4-mediated ROS production [79].

Similar to extrinsic apoptosis, necroptosis involves the interaction between death receptors TNFR of FsR with their ligands and leads to cell death by a caspase-independent mechanism [80]. Necroptosis implies the autophosphorylation of receptor-interacting serine/threonine-protein kinase 1 (RIPK1), followed by the recruitment and autophosphorylation of RIPK3 to form a heterodimeric complex. Then, RIPK3 phosphorylates the Mixed Lineage Kinase Domain-Like Pseudokinase (MLKL), which induces its oligomerization and the formation of membrane-disrupting pores. It has been demonstrated that mitochondrial ROS promote RIP autophosphorylation [81], and at the same time, the formation of RIPK1/RIPK3 complex augments ROS accumulation, creating a positive feedback loop. Growing evidence suggests a clear association between ROS and necroptosis useful in cancer treatments, in particular for bypassing apoptosis and triggering cell death through an alternative mechanism in apoptosis-resistant cancer cells [82,83,84,85].

Lastly, ROS is also involved in ferroptosis, another form of regulated cell death driven by iron-dependent phospholipid peroxidation. This process is catching the interest of researchers since drug-resistant cancer cells seem to be particularly susceptible to this type of programmed cell death. In this case, excessive ROS-induced lipid peroxidation and mitochondrial dysfunction can initiate ferroptosis and consequent cell cancer death. In the last few years, it has been observed that in transferrin receptor overexpressed human colorectal adenocarcinoma grade II cell line HT29, Doxorubicin-loaded ferritin nanoparticles induce the ferroptosis process through a ROS-dependent mechanism in parallel to a declined activity of GPX4 [86]. Very recently, the therapeutic role of Sodium Butyrate (NaBu), the sodium salt of butyric acid, in the progression of endometrial cancer has been demonstrated. NaBu increases ROS levels and promotes ferroptosis in endometrial cancer cells by inhibiting the expression of SLC7A11, a cystine-glutamate exchanger that takes up cystine into the cell, inducing the synthesis of GSH, which, in turn, enhances the antioxidant cellular system and inhibits ferroptosis. SLC7A11 exerts tumor-promoting effects in different human cancers [87,88]. This result is very encouraging since it has been observed that gut microbiota plays an important role in the development and progression of many human cancers. Butyric acid is a short-chain fatty acid produced by gut microbiota; therefore, it could be considered an interesting molecule for the development of therapies aimed at reducing tumor metastasis just through the ROS-dependent process of ferroptosis [89].

As we have discussed so far, ROS plays a key role in a variety of cancers; at this point, our review will mainly focus on melanoma as it has been defined as one of the most representative “ROS-driven tumor” [6]. 

## 6. Melanoma Characteristics and Pathophysiology

Cutaneous melanoma (hereafter, melanoma) is a malignant tumor arising from melanocytes, the pigment-forming cells of the skin. Although melanoma accounts for only 1% of all skin cancers, it is a very aggressive pathology and the main cause of skin cancer-related mortality [90]. The incidence of melanoma is increasing worldwide, and it has been projected that the number of new cases will rise by more than 50% in the next twenty years. There is a large geographical variation in melanoma incidence across world regions, with the highest occurrence in Australia/New Zealand, followed by Western Europe, North America, and Northern Europe, and the lowest in most African and Asian countries [91]. 

Most melanomas appear to arise de novo; indeed, especially in young people, the risk of a nevus becoming malignant is very low, between 0.0005% and 0.003%, as reported in the population-based estimate by Tsao and colleagues [92]. 

Melanoma is considered a multifactorial human cancer, and risk factors for this pathology can be divided into host, genetic, and environmental; first, the frequency of melanoma is higher in light-skinned (phototype I and II based on the Fitzpatrick classification) than in dark-skinned individuals (phototype V and VI based on the Fitzpatrick classification). Concerning family history, familial melanoma accounts for about 10% of all diagnosed cases of cancer; in particular, the predisposition for hereditary melanoma has been ascribed to a mutation in one of the defined high-penetrance predisposition genes (viz., cyclin-dependent kinase inhibitor 2A (CDKN2A), cyclin-dependent kinase 4 (CDK4), BRCA1-associated Protein 1 (BAP1), Protection of Telomeres 1 (POT1), adrenocortical dysplasia (ACD), telomerase reverse transcriptase (TERT), and telomeric repeat-binding factor-2 interacting protein (TERF2IP)) [93]. 

Like other tumors, the patterns of genetic alterations in melanomas suggest that the starting point of neoplastic proliferation is the accumulation of the gain-of-function mutations of genes involved in cell growth. This can occur through point mutations, gene fusions, and gene amplification [94]. 

## 7. Mutated Driver Genes and Downstream Signal Pathways Involved in Melanoma Progression

Regarding somatic mutations, melanomas are divided into four subtypes: (1) B-Raf proto-oncogene serine/threonine kinase mutant (BRAF), (2) NRAS proto-oncogene GTPase mutant (NRAS), (3) neurofibromin 1 mutant (NF1), and (4) triple wild-type (WT) BRAF/NRAS/NF1 [95]. In detail, about 50% of melanomas are BRAF mutations (principally mutations at the V600 codon), around 30% are RAS mutations, 10–15% are NF1 mutations, and about 5–10% are triple wild-type mutations. Of note, all these mutations are, in turn, responsible for the hyper-activation of the MAPK pathway and, consequently, for augmented cell proliferation. Besides MAPK, multiple signal pathways involved in cell cycle progression are altered during melanoma progression, including (1) the PI3K signaling cascade and its negative regulator tumor suppressor phosphatase and PTEN [96], (2) CDKN2A, which encodes the two tumor suppressor proteins, p16 (also known as p16^INK4a^) and p14 (also known as p14^ARF^) [97], (3) microphthalmia-associated transcription factor (MITF), a key transcription factor for melanocyte development and differentiation [98], (4) the master regulator of the pro-inflammatory gene expression program, NF-κB [99], and (5) the cyclin D1 and CDK4/6 [100,101]. 

Regarding the genetic factors related to melanoma, the polymorphisms of the melanocortin 1 receptor (MC1R) gene are associated with cancer development [102] MC1R is a G protein-coupled receptor characterized by seven transmembrane segments, an extracellular N-terminus, and an intracellular C-terminus located on the cell surface of melanocytes [103]. MC1R has a pivotal role in pigmentation, although more recently, it has also been demonstrated its non-pigmentary function as a regulator of antioxidant cellular defenses and DNA-repair mechanisms [104]. The binding of α-melanocyte stimulating hormone (α-MSH) to MC1R prompts the activation of adenylyl-cyclase (AC) activity and a consequent increase in intracellular cAMP levels. In turn, the augmented cAMP level switches melanin production from the red/yellow pheomelanins to the brown/black eumelanins [105]. Variants in MC1R are not able to activate this signaling cascade, and pheomelanogenesis is preserved. Since, compared with eumelanin, pheomelanin has a weak shielding capacity against ultraviolet (UV) radiation harmful effects, MC1R variant carriers are commonly associated with increased susceptibility to develop melanoma, as described subsequently [100]. Moreover, mutations in DNA repair genes (viz., the genetic disorder xeroderma pigmentosum gene (XP) [106]) and cell cycle-regulated genes (CDKN2A, CDK4 [107]) correlated with enhanced risk of melanoma development.

## 8. Melanoma Redox Regulation

Oxidative stress and ROS production are involved in all phases of melanoma development and progression as well as in the drug resistance process [108,109], and indeed, it is defined as a “ROS driven tumor” [6]. 

The growth incidence of melanoma is expected to be due to a specific environmental risk factor: increased exposure to UV radiation from any sources, natural and artificial. It has been estimated that more than three-quarters of all newly diagnosed melanoma cases can be ascribed to UV radiation [101]. The UV radiations, the invisible part of light spectra, are divided into UVA (315–400 nm), UVB (280–315 nm), and UVC (100–280 nm). Two types of UV radiation are principally involved in melanoma development and progression: UVA and UVB. Indeed, UVC, despite having the higher energy, is completely absorbed by stratospheric ozone. It needs to be mentioned that 95% of UVB rays are mostly absorbed by the ozone layer, so the ultraviolet radiation that reaches the Earth’s surface is mainly composed of UVA, with a ratio of 95% UVA and 5% UVB [110]. 

It is consequently evident that the ozone layer acts as a natural filter; therefore, its progressive depletion due to industrialization, air pollution, and global warming is associated with an increase in UV radiations reaching the Earth’s surface, causing an increase in melanoma incidence estimated around 4% to 5% annually [111]. UVA and UVB differ from each other since although UVB has higher energy than UVA, UVA penetrates deeper into the different layers of the skin. Despite this difference, both UVA and UVB have harmful effects on human health by targeting specific biomolecules such as DNA, proteins, and lipids, altering their structure and functions [112,113]. 

Other cellular non-DNA chromophores in the different skin layers are urocanic acid, riboflavins, haem, bilirubin, porphyrins, melanin precursors, pterins, flavins, carotenoids, and chromophoric amino acids such as tryptophane, tyrosine, phenylalanine, histidine, and cysteine [114]. The skin non-DNA chromophores function as photosensitizers involved in skin photoaging and photocarcinogenesis. Indeed, after the absorption of UV photons, the formation of photoexcited states of these molecules induces the generation of ROS and other toxic photoproducts that, themselves, propagate the photochemical damage to DNA and to the other biomolecules of the skin [115,116,117]. Moreover, the UV radiations can indirectly lead to the production of ROS activating specific cellular enzymes, such as COX, NOX, and xanthine oxidase, or by the involvement of mitochondria [113], as mentioned before. 

Besides the above described exogenous and endogenous sources that can alter the redox status of skin cancer cells, another source of ROS in melanoma is the process of melanin biosynthesis. 

Data from the literature strongly support the photoprotective role of melanin against UV radiation [118]. The skin melanocytes synthesize two types of melanin: brown-black eumelanin and red pheomelanin. Pigments formed in melanosomes are then transported to neighboring keratinocytes, where they offer photoprotection. Indeed, melanin can act as a UV filter to prevent direct UV action on the DNA of skin cells. As described by Gloster and Neal, eumelanin is thought to have more photoprotective properties than pheomelanin [119]. As early as a couple of decades ago, it has been shown that while dark skin (phototype IV–VI) allows only about 8% and 18% of UVB and UVA penetration, respectively, about 25% of UVB and 55% of UVA are able to penetrate through light skin (phototype I–III) [120].

However, melanin can also have toxic properties, mainly upon UV exposure. Indeed, melanin biosynthesis implies a sequence of oxidation reactions involving tyrosine catalyzed by tyrosinase with the generation of O_2_^−^ and H_2_O_2_, which expose melanocytes to oxidative stress [121]. Therefore, the biosynthesis of melanin itself induces ROS production in melanocytes [122].

Noonan and colleagues [123] demonstrated that melanoma initiation by UVA in pigmented (>90% eumelanin) or albino mice differed considerably, suggesting that melanin pigment is associated with a higher probability of developing the tumor. The authors also show that, in contrast, UVB radiation induces melanoma in a pigment-independent manner, which is related to a direct effect on DNA damage. Conversely, recently, an inhibitory effect of the pigment on melanoma metastasis was demonstrated [124]. In this elegant work, the authors inoculated in nude mice human melanoma SKMEL-188 cells with different amounts of melanin, and they demonstrated that the cancer cells with a higher melanin amount formed an equal volume of tumor, although it looks more compact with a regular border when compared to tumor formed by amelanotic cells. This result confirmed that the presence of melanin makes melanoma cells less prone to spread. 

As reported before, the altered redox status that characterized melanoma is strongly due to the activity of different enzymes such as NOXs, COX, lipoxygenases (LOXs), or other ROS-producing molecules that activate specific signal transduction pathways [125]. 

Among NOX enzymes, NOX1, NOX4, and NOX5 are expressed in melanocytic lineage [125]. NOX1 is expressed in normal melanocytes and in melanoma cell lines, including the early radial growth phase cell line Wm3211. However, the protein level does not correlate with melanoma progression, suggesting that its upregulation is an early event in melanoma transformation. NOX4 is expressed in only a subset of metastatic melanoma cell lines, implying that it is required for ROS-mediated metastatic processes [126]. Govindaraja et al. [127] suggested that NOX4 is involved in the progression of human melanoma, demonstrating that NOX4-generated ROS activated by Akt enabled the conversion of radial growth to vertical growth essential for the invasive and metastatic phenotype. Indeed, only the expression level of isoform 4 is higher in metastatic tumors compared with primary melanoma, confirming a potential role of ROS generated by NOX4 in transmitting cell survival signals among melanoma cells [128,129]. Recently, the correlation of NOX4 with another pathway involved in the different stages of melanoma, the hepatocyte growth factor (HGF)/c-met axis, has been analyzed. It has been observed a correlation between the expression level of both c-met and NOX4, indicating them as possible melanoma severity markers, as well as potential cellular markers for a therapeutical strategy to treat this cancer [130]. Finally, NOX5 is also overexpressed in melanoma cells, and it seems to be involved in cell proliferation through the ROS-mediated hypoxia-inducible factor (HIF)-1α and p27Kip1 signaling pathways [131]. 

While oxidative stress drives melanoma development, causing oncogenic mutations and activating oncogenic pathways, it is considered a double-edged sword during melanoma progression. Searching the PubMed database with “reactive oxygen species” and “melanoma” returned about 2000 publications from 1977 and 2024. While different papers demonstrated the harmful effects of ROS in melanoma etiology and progression, many others suggest tumor suppressive functions of ROS due to their pro-apoptotic role: this discrepancy makes it difficult to draw any conclusions. Despite the divergent results obtained in numerous in vitro studies conducted on melanoma cell lines, the data that emerged from in vivo experiments are also unique. Piskounova and colleagues published very exhaustive data on the role of oxidative stress on melanoma-distant metastasis [132]. The authors analyzed melanomas from several patients that were xenografted into NSG mice. In their experiments, melanoma metastasis was predictive of clinical outcomes in patients. They found that oxidative stress both limits tumorigenesis by circulating melanoma cells and confines the metastasis in vivo. On the other hand, Sander and colleagues [133], using an immunohistochemical approach, found an increase in lipid peroxidation products (LPOs) in human melanoma biopsies when compared to benign melanocytic naevi and healthy controls. Moreover, they also observed high LPO levels in surrounding keratinocytes, indicating that generalized oxidative damage might be a mechanism of melanoma cells to promote metastatic processes. Confirming the difficulties in drawing a clear picture of the role of oxidative mediators in melanoma progression and metastatic properties. 

## 9. Nrf2 and NF-kB: The Two Key Factors Involved in Melanoma Redox State

What is unambiguously accepted is that melanoma cells exhibit a greater ROS level than normal cells. Excessive ROS production in melanoma can affect tumor progression through the regulation of different signaling pathways involved in abnormal cell proliferation and metastasis. It should be mentioned that the PI3K/Akt/mammalian target rapamycin (mTOR), the mitogen-activated protein kinase (MAPK), NF-κB, and Nrf2 pathways [134]. The PI3K/Akt/mTOR pathway is deregulated in different human cancers, and its overactivation promotes cell survival and inhibits apoptosis [135]. ROS activates this pathway in melanoma to promote proliferation and migration. There is extensive literature on the regulation of these cellular mechanisms by ROS, and we believe that two interconnected signaling pathways that are worthy of more detailed description are those regulated by the two redox-sensitive transcription factors, Nrf2 and NF-κB [136].

Indeed, Nrf2 is a key transcriptional regulator of the oxidative stress response, and its crosstalk with NF-κB is crucial in the regulation of cellular redox homeostasis. The reduced activation of Nrf2 enhances NF-κB activity, whereas NF-κB can regulate Nrf2 transcriptional activity positively or negatively. Then, these two transcription factors cooperatively maintain cellular homeostasis, although, under different conditions, they can promote carcinogenesis.

### 9.1. Nrf2 and Oxidative Stress

Nrf2 is a note transcription factor that controls cell homeostasis against oxidative and toxic insults, mediating the transcription of a wide array of genes involved in the antioxidant cellular response [137]. Under homeostatic conditions, Nrf2 activation is suppressed by its interaction with Keap1, an adaptor subunit of Cullin 3-based E3 ubiquitin ligase harboring 27 cysteine residues. The Keap1–E3 ubiquitin ligase complex tightly controls Nrf2 to keep it at a low level by targeting its ubiquitination and proteasomal degradation in the cytoplasm. Under oxidative stress, the oxidation of specific Keap1-cysteines consents Nrf2 to avoid ubiquitination and to translocate into the nucleus. In the nucleus, it forms a heterodimer with small Maf (musculoaponeurotic fibrosarcoma) proteins (Maf-F, Maf-G, and Maf-K), which are necessary for the Nrf2-related upregulation of antioxidant response element (ARE)-dependent target genes [138]. These genes encode for phase II antioxidant enzymes such as glutathione synthetase (GSS), glutathione reductase (GR), Gpx, Trx, thioredoxin reductase (TRR), and peroxiredoxin (PRX) involved in cell protection against oxidative stress. Specifically, the increased level of intracellular ROS enhances the activation of Nrf2, promoting the dissociation between Nrf2 and Keap1 through the oxidization of specific Keap1 cysteine residues (Cys273, Cys288, and Cys151). The phosphorylation of Nrf2 has also been proposed as an alternative process for escaping Keap1-mediated repression. Indeed, ROS can activate specific cellular kinases, such as protein kinase C (PKC), MAPK, PI3Ks, and protein kinase-like endoplasmic reticulum kinase (PERK), which, in turn, phosphorylate Nrf2, at the level of a single serine residue, S40 [139,140,141].

Nrf2 has been conventionally thought as a major regulator of cell survival, having a key role also in preventing various oxidative stress-related pathologies [142], such as aging, Alzheimer’s and other neurodegenerative diseases [143], respiratory diseases (asthma, chronic obstructive pulmonary disease bronchopulmonary dysplasia, respiratory infections, acute respiratory distress syndrome, idiopathic pulmonary fibrosis) [144], diabetes [145], diseases of the gastrointestinal tract, viz., inflammatory bowel diseases [146], and many others. Despite this, it is now well known that Nrf2 is also associated with the progression of different human cancers, including melanoma, to the extent that it is considered a key transcription molecule in melanoma redox manipulation. Overall, it can be assumed that the onco-promoter/onco-suppressor role of Nrf2 varies by melanoma stage, and whereas transient Nrf2 activity protects against tumor development, constitutive Nrf2 activation may encourage cancer progression and metastasis. Nevertheless, the scientific world is still divided on the exact role of this ROS-activated transcription factor in the different stages of melanoma progression.

### 9.2. The Role of Nrf2 in Melanoma Development

An immunohistochemical/immunochemical study conducted on 255 samples obtained from 172 patients with different diagnoses of melanoma (nevi, primary melanomas, and melanoma metastases) showed how Nrf2 expression decreases very early in melanoma carcinogenesis process, while its expression levels increase from primary to metastatic lesions. Moreover, the authors observed that the decreased Nrf2 nuclear expression level is an indicator of reduced patient survival [147]. Therefore, this study suggests that Nrf2 has a protective role against melanoma development but can be considered a tumor-progressing factor in the malignant phases of this tumor.

**Figure 2:**
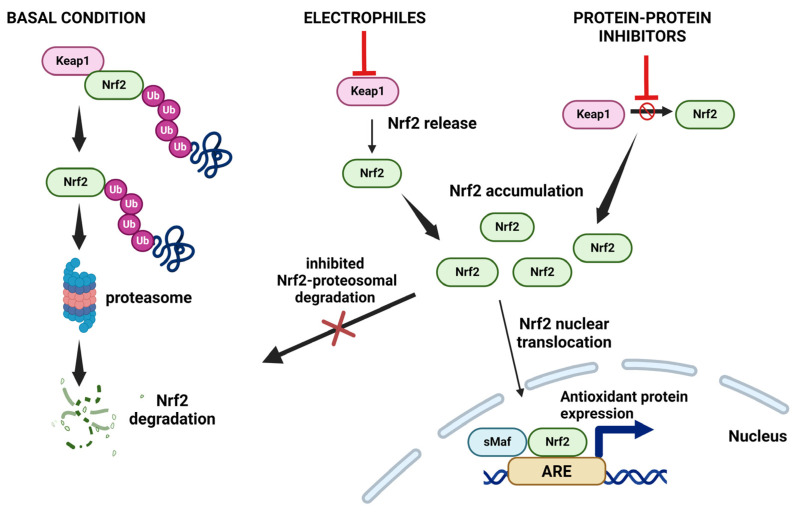
Electrophiles and protein–protein inhibitors modulation of the Keap1–Nrf2 system. Left panel: Under basal conditions, Nrf2 is constantly ubiquitinated through Keap1 and degraded in the proteasome. Right panels: When exposed to electrophiles (Keap1 inactivated) or protein-protein inhibitors (Keap1–Nrf2 binding inhibited), stabilized Nrf2 accumulates in the nucleus and activates transcription of antioxidant genes. Ub, ubiquitin. Image created with Biorender.com.

In a very interesting paper [148] using SKH-1 hairless mice in which Nrf2 is constitutively activated, the authors demonstrated that genetic activation of Nrf2 prevents skin carcinogenesis due to UV radiation. Moreover, they showed that in healthy human subjects, upon acute exposure to UV radiation, the topical application of broccoli (*Brassica oleracea*) extracts containing the Nrf2 activator sulforaphane reduces the degree of skin erythema, a clear risk for melanoma development. Therefore, they speculated that Nrf2 cannot be considered a driver for the development of skin cancer. Since Nrf2 has a protective role in healthy human skin, its activation usually should be favored in preventing melanoma development. The pharmacological activation of Nrf2 can be achieved using natural compounds or synthetic drugs that modulate the Keap1–Nrf2 system. In spite of their nature, they can be classified as electrophiles or Keap1–Nrf2 protein–protein interaction (PPI) inhibitors [149]. The electrophilic compounds (such as sulforaphane, curcumin, resveratrol, quercetin, and genistein) oxidize specific cysteine residues of Keap1 (especially Cys-151, Cys-273, and Cys-288), leading to Keap1 inactivation and the impairment of its capability to target Nrf2 for proteasome degradation. Therefore, the ex novo synthesized Nrf2 can migrate into the nucleus and promote the transcription of target genes (Figure 2).

On the other hand, the PPI inhibitors of the Keap1–Nrf2 system prevent the docking of Nrf2 to Keap1, with a mechanism of action that is more selective than that of the electrophilic compounds (Figure 2). A clear and detailed description of both classes of compounds was summarized in the recent review of Natalia Robledinos-Antón and colleagues [149].

### 9.3. The Role of Nrf2 in Melanoma Progression

While Nrf2 has a protective role against melanoma carcinogenesis, it can be considered a tumor-progressing factor in the malignant phase [147]. Indeed, when melanoma progresses in such a way that cell transformation occurs, metabolic reprogramming results in constitutive activation of Nrf2 and its downstream enzymes. Consequently, the cancer cells use Nrf2 to protect themself, and its higher expression makes tumor cells resistant to high levels of ROS produced by cell metabolism. In this regard, Nrf2 is associated with invasiveness and pro-metastatic features.

The tumor-promoting role of ROS mediated Nrf2 activation in melanoma progression has been frequently attributed to its correlation with MAPK signaling. As mentioned before, in melanomas, tumor transformation is frequently determined by the dysregulation of the MAPK pathway because of BRAF and NRAS mutations. Interestingly, the crosstalk between Nrf2 and MAPK signaling pathways has been suggested to be both beneficial and unfavorable for melanoma progression. Indeed, Takasaki and colleagues have demonstrated that ACA-28, a novel anti-cancer compound, induces ERK-dependent apoptosis in melanoma cell lines in a ROS-dependent manner. Moreover, ACA-28, via its ability to stimulate ROS production, activates Nrf2 signaling, which in turn protects cancer cells from ACA-28-mediated cell death, endowing their resistance to this specific agent. Finally, to maintain the effectiveness of this compound, the authors combined ACA-28 with a specific inhibitor of Nrf2 (ML385), demonstrating the efficacy of this combination in inhibiting melanoma cancer cell viability [150]. In addition, Yu and colleagues confirmed the pro-tumorigenic role of Nrf2, showing how tetrahydroisoquinoline alkaloids, renieramycin T inhibit B16F10 mouse melanoma cells migration and invasion, probably through the phosphorylation of the Signal Transducer and Activator of Transcription 3 (STAT3) and the downregulation of Nrf2, suggesting their crosstalk in melanoma progression [151]. On the other hand, it has been demonstrated that delphinidin, potent anthocyanidins in berries, inhibited the TPA-induced neoplastic cell transformation of mouse epidermal JB6 P+ cells by inducing the activation of Nrf2 and its target genes involved in phase II antioxidant cell response (i.e., HO-1), suggesting delphinidin as a potential skin cancer chemopreventive molecule thanks to its ability to activate Nrf2-ARE pathway [152].

In another work, Nrf2 was presented as an antitumoral molecule that reduces melanoma growth and metastasis. In Nrf2-null C57BL/6 mice inoculated with B16-F10 melanoma cells, a remarkable increase in tumor growth and lung metastasis was observed as compared with wild-type mice. Although this is not a mechanistic paper, the authors suggested that this effect could be due to the dysregulated immunity in Nrf2-null mice [153].

Recently, it was shown, even if indirectly, that sesquiterpenes lactones selected cynaropicrin, isolated from the aerial parts of *Centaurea drabifolia* subsp. *detonsa*, reduce the proliferation, migration, invasion, and clonogenic ability of human metastatic BRAF mutant melanoma cells, also mediating the apoptosis process. The authors also demonstrated that in A375 cells, cynaropicrin reduces the MAPK and NF-κB pathways and, at the same time, augments endogenous antioxidant properties (an increase in Nrf2 activity and expression of its target genes), as well as decreasing the intracellular ROS generation [154]. This anti-oncogenic role of Nrf2 could also be explained by virtue of its crosstalk with the abovementioned ROS-regulated transcription factor NF-κB. 

### 9.4. The Role of Nrf2 in Melanoma Resistance to Immunotherapy and Targeted Therapy 

Although the treatment of metastatic melanoma has been implemented by improvements in immunotherapy (viz., an anti-programmed cell death protein 1 (PD-1), anti-programmed death ligand-1 (PDL-1), cytotoxic T-lymphocyte-associated protein 4 (CTLA-4)) and targeted therapy (viz., BRAF and MEK Inhibitors) [155], unfortunately most patients develop therapeutic resistance associated with cancer progression and low survival rates [156]. A role of Nrf2 in both immuno- and targeted therapy resistance has been documented; specifically, regarding immunotherapy, Nrf2 may modulate the innate immune responses, mainly in advanced stages of melanoma, suppressing the pro-inflammatory mediators’ expression through the activation of antioxidant genes [157]. In a recent study, it was demonstrated that in murine B16 melanomas, the stable NRF2 silencing by a specific shRNA knockdown induces the PD-1/PD-L1 inhibition to activate infiltration T cells and the reduction of melanoma growth [158]. Moreover, as described by Carpenter and colleagues, Nrf2 could be connected to resistance to immunotherapy since it negatively regulates the nuclear receptor, retinoid X receptor α (RXRα) expression, a member of the nuclear hormone receptor superfamily, known as central coordinators of cell signal transduction [159]. Indeed, the authors showed that the loss of RXRα in melanocytes leads to decreased recruitment of IFN-γ-secreting immune cells following UV exposure [160]. Then, they suggested that since IFN-γ positively regulates PD-L1 expression and higher PD-L1 expression has been correlated with the improved efficacy of anti-PD-1 therapy, the Nrf2 negative regulation of RXRα could be connected to treatment resistance. 

Moreover, the role of Nrf2 in target therapy resistance has also been suggested; recently, a key role of Nrf2 in melanoma BRAF/MEK inhibitor resistance has been demonstrated. First, the authors found that Nrf2 is upregulated in melanoma cells resistant to targeted therapy, and then they verified that the inhibition of the transcription factor reverted the resistance to BRAF/MEK inhibitors [161]. Khamari and colleagues also demonstrated that BRAFi-resistant melanoma displays a robust activation of Nrf2 and consequent metabolic modifications that induce an increase in glutathione level that, in turn, promotes the intracellular redox balance that allows for the survival of BRAFi-resistant melanoma cells [162].

### 9.5. NF-κB and Oxidative Stress

NF-κB family comprises five different proteins: RelA, RelB, c-Rel, p105/p50, and p100/p52, which are characterized by an N-terminal Rel homology domain (RHD), which is required for dimerization and DNA binding. RelA, RelB, and cRel also contain transcriptional transactivation domains that induce the expression of different genes involved in cell proliferation and apoptosis. RelA, cRel, p105/p50, and p100/p52 can form homo- and hetero-dimers. Upon activation, these dimers translocate into the nucleus, where they bind to NF-κB sequences, kB enhancers, on DNA, mediating the transcription of specific target genes. Normally, NF-κB is trapped in the cytoplasm by the IkB inhibitory protein, but upon different stimuli, it dissociates from IkB and translocates into the nucleus, where it completes its transcriptional activity [163]. 

Concerning oxidant-induced NF-κB activation, in late 1991, Schreck et al. first demonstrated that the addition of H_2_O_2_ to the culture medium of Jurkat cells could activate NF-κB [164]. Then, different research groups focused on the study of this correlation, demonstrating that NF-κB activation by H_2_O_2_ is cell-type specific and implies different molecular mechanisms. While, classically, IκBα is phosphorylated on serines 32 and 36, leading to its ubiquitination and degradation, H_2_O_2_ induces the phosphorylation of IκBα on Tyr42 or other tyrosine residues, and it may or may not be degraded [165,166]. In this case, although IKK is also phosphorylated, it is not fundamental, and IκBα phosphorylation may be mediated by casein kinase II. Moreover, the degradation of IκBα may not be necessary in this case since Tyr42-phosphorylated IκBα is bound by the SH2 domains of the p85α regulatory subunit of PI3K, exposing NF-κB and allowing it to translocate into the nucleus [167]. In other cases, H_2_O_2_ can directly modulate IKK, as finely described by Morgan and Liu [168]. As the authors concluded, ROS may interact with the NF-κB signaling cascade in different ways in fine-regulated crosstalk that appears to be cell-type specific. Indeed, the transcription of NF-κB-dependent genes controls the cellular ROS levels, but at the same time, also the levels of NF-κB activity are, in turn, adjusted by the levels of ROS. 

### 9.6. NF-κB in Melanoma

As described, ROS enhances the signal transduction pathways of NF-κB and, since melanoma is a human cancer with a constitutive oxidative stress status, unsurprisingly, as in another human tumor, the activation of NF-κB is unambiguously considered a hallmark of tumor development and progression. By exploring the literature, we can find many papers asserting that the activation of NF-κB is an event that promotes melanoma tumor progression, but only limited data focused on its role in cancer development. The group of McNulty and colleagues showed both in cell cultures and in human tissue biopsies that NF-κB is constitutively elevated in human metastatic melanoma cells compared with normal melanocytes [169,170]. In this immunohistochemical study, the authors analyzed 60 human biopsies derived from normal skin, benign naevus, and metastatic melanoma. They found not only higher expression of RelA in melanoma cells of patient biopsies than in melanocytes found in normal epidermal tissues, but they also observed that RelA expression is significantly higher in the melanocytes found in benign intradermal naevus biopsies compared with melanocytes found in normal epidermal skin tissues. Therefore, they speculated that deregulation in RelA expression may be correlated with tumors that arise from a naevus but not those that occur independently [171]. As mentioned above, the notions that NF-κB activation is an event that promotes melanoma progression and that ROS-mediated increases in NF-κB sequentially drive the antiapoptotic process have long been known; NF-κB promotes the upregulation of antiapoptotic proteins just detectable in melanocytic nevi, and that increases during melanoma progression and metastasis; between them, there is the tumor necrosis factor receptor-associated factor 1 and 2 (TRAF1, TRAF2), c-IAP1, c-IAP2 proteins, the melanoma inhibitor of apoptosis (ML-IAP), and Bcl-2 proteins, as mentioned above [172,173]. In addition, to foster melanoma cell proliferation, NF-κB activation may control the expression of cell cycle regulatory proteins such as cyclin D1 and CDK2. Their overexpression enables melanoma cells to escape the cell-cycle control mechanisms contributing to tumor growth [174,175]. Finally, the constitutive activation of NF-κB in melanoma cells also drives the augmented expression of endogenous chemokines, such as interleukin (IL)-1, IL-6, IL-8, and vascular VEGF, which, when transcriptionally stimulated, are assumed to increase melanoma progression [99].

However, the antiapoptotic role of this redox-sensitive transcription factor is also widely discussed in the literature, and here we report some of the more recent results. Over the past few years, different studies focused on the study of specific compounds that, while increasing ROS levels, at the same time, hinder the activation of NF-κB and other pro-survival pathways or the activation of pro-apoptotic processes. 

Several research groups analyzed the effect of natural compounds that could be used as adjuvants to conventional therapies, with the aim of reducing their side effects. 

Last year, Cardile and colleagues published a very exhaustive paper on the role of Hyperforin in the progression of different human metastatic melanoma cell lines. Nowadays, Hyperforin from *Hypericum perforatum*, besides its antidepressant action, is also considered for its anti-inflammatory, antimicrobic, and antitumor activities. In melanoma cells, Hyperforin downregulates several cytosolic (phosphoglucomutase 2 (PGM2), lactate dehydrogenase A (LDHA), and the phosphorylated form of pyruvate kinase M2 (pPKM2)) and mitochondrial (ubiquinol cytochrome c reductase (UQCRC1), cytochrome c oxidase subunit IV (COX4), and ATP synthase F1 subunit β (ATP5B)) enzymes, indicating a generalized decrease of metabolic functions. In addition, Hyperforin increasing ROS cellular level induces a reduction in melanoma cell proliferation by affecting different pathways, among them NF-κB, hindering its activation [176]. In another work, the role of ROS-induced NF-κB in melanoma progression was verified in a co-culture system of B16 melanoma cells and RAW 264.7 macrophage-like cells to assess the function of *Tremella fuciformis* polysaccharides (TFPSs) as a possible immunomodulatory molecule for melanoma therapy. First, the authors demonstrated that TFPS increases the apoptosis rates of B16 cells in this co-culture system, mimicking the tumor microenvironment. Moreover, they demonstrated that TFPS may promote apoptosis of B16 cells by polarizing tumor-associated macrophages (TAMs) to a pro-inflammatory (M1) phenotype that promotes immune responses to tumors and is characterized by increased ROS level and alteration of MAPK and NF-κB pathways [177]. 

Recently, interesting data has been obtained on the anti-melanoma effects of essential oils obtained from aerial parts of *Conyza bonariensis* (CBEO), a common weed in South America. First, Ferreira et al. [178] demonstrated that CBEO in the SK-MEL28 malignant melanoma cell line increases the intracellular ROS level, inducing also a reduction in cell viability. Then, the same research group showed how this effect was mediated by the alteration of intracellular signaling pathways, including MAPKs and NF-κB. Interestingly, the ROS-dependent apoptosis of SK-MEL28 cells upon CBEO treatment is associated with the activation of NF-κB. Consequently, in this case, the upregulation of NF-κB sensitizes cancer apoptosis, underlining how its role in melanoma progression is very complex to understand [179]. This double role of NF-κB was just reviewed by Perkins et al. [180] and observed in other human cancers [181]. In general, such as in other human cancers [182], in the initial stages of melanoma, NF-κB might be considered a tumor-suppressor rather than a tumor-enhancer, while during melanoma progression, there is a reversal of its role, and NF-κB promotes the expression of a wide range of genes involved in tumor malignant progression.

### 9.7. The Role of NF-κB in Melanoma Resistance to Immunotherapy and Targeted Therapy 

Besides the above-described role of NF-κB as a promoter of abnormal cancer cell division and survival, it could contribute to melanoma progression through the activation of immune checkpoints, such that it is considered a molecular target in immunotherapy approaches. The direct involvement of NF-κB in melanoma response to immunotherapy was recently confirmed using a multi-scale network approach to discover gene modules with coordinated gene expression upon the treatment with the immunotherapy drug nivolumab [183]. Differently from Nrf2, the role of NF-κB in immunotherapy resistance is more complex due to its modulation of the function of immune cells that populate the tumor environment, impacting cancer outcomes. For a better understanding of this issue, we refer to a seminal review by Lalle and colleagues, which summarized the multifaceted roles of NF-κB in orchestrating tumor immunity and in modulating the immunotherapeutic efficacy in different human cancers, including melanoma [184].

Moreover, the activation of NF-κB, alongside low activity of MITF, are considered hallmarks of melanoma resistance in patients treated with BRAF/MEK inhibitors [185]. Specifically, these analyses suggested that a high NF-κB state is associated with reduced sensitivity to MAPK pathway inhibition with specific drug treatment, suggesting a key role of the NF-κB in inducing the target therapy resistance [186].

Based on this assumption, it has recently been shown that the downregulation of miR-146a observed in six melanoma cell lines with acquired resistance and in two lines derived from drug-resistant tumors could be considered an epigenetic mechanism involved in the maintenance of the high level of NF-κB associated to target therapy resistance due to its negative regulation of this transcription factor [185].

### 9.8. Nrf2-NF-κB Crosstalk in Melanoma Progression

Since we discussed the role of ROS-driven Nrf2 and NF-κB activation in human melanoma and their dual function as pro- and anti-oncogenic molecules, we believe that it is appropriate to better dissect the interplay between them. Nrf2 inhibits NF-κB nuclear translocation by decreasing the intracellular ROS level and inducing the activation of the phase II enzyme Heme Oxygenase-1(HO-1), which is able to reduce cellular inflammation [187,188,189,190]; HO-1 prevents the IκB proteasomal degradation and consequent NF-κB nuclear translocation/activation via its end-products (carbon monoxide and bilirubin) [191]. At the same time, NF-κB was also shown to prevent the transcription of Nrf2-dependent genes, reducing the free CREB-binding protein (CBP), a transcriptional activator of Nrf2 itself [192]. Indeed, both p65 and Nrf2 bind to the CH1-KIX domain of CBP, and the binding of either transcription factor depends on their relative amount into the nucleus (viz., on their activation) [193]. Moreover, p65 promotes the recruitment of HDAC3, a corepressor, facilitating the interaction of HDAC3 with either CBP or MafK, leading to local histone hypoacetylation and impeding Nrf2 signaling [182]. Starting from this evidence, it becomes clear how the elevated oxidative stress that characterizes human melanoma progression can enhance Nrf2 level and activate NF-κB, which could eventually prevent Nrf2 activation, leading to a vicious circle where the increase in NF-κB activation could promote an anti-or pro-apoptotic effect depending on the malignancies stage. At the same time, the increased ROS levels could activate Nrf2 and prevent NF-κB activation, leading to a further increase in Nrf2 levels and implementing cell survival.

Therefore, considering their mutual modulation, the development of a possible target therapy focused on this complex crosstalk may not simply be reduced to an antioxidant versus pro-oxidant option but must be carefully adjusted according to the stage of the cancer.

Indeed, while exogenous antioxidants may represent an important strategy in melanoma prevention, they do not have a clear therapeutic role in the progression of this human cancer since they generate an advantageous environment that supports the survival of cancer cells by protecting them from oxidative stress, chemotherapeutic drugs, and radiotherapy. 

The results from the literature regarding the advantages of both anti- and pro-oxidant therapies are far from unique and will be discussed in the next paragraph.

## 10. Antioxidant vs. Pro-Oxidant Therapeutic Approach: What Is More Effective?

Nowadays, the most common therapeutic strategies for human melanoma comprise surgical approaches, with wide excision margins of the primary tumor, and non-surgical approaches, such as radiation, chemotherapy, immunotherapy, and targeted therapy [194,195,196]. The surgical approach is useful and curative in primary melanoma, localized to the skin, but unfortunately, it is not curative in case of metastasis when the tumor is difficult to manage and causes a high mortality rate. In case of multiple metastatic tumors, other therapeutic approaches are favored; radiation therapy has a restricted usage and is recommended in case of metastasis difficult to reach; systemic therapies are considered for the advanced stages of melanoma, and nowadays, the first untargeted chemotherapies (e.g., dacarbazine and temozolomide) have been substituted by more effective targeted therapy and immunotherapy [195]. Despite this wide range of systemic approaches for metastatic melanoma, their effectiveness is incomplete due to the development of drug resistance [197]. 

### 10.1. Pro-Oxidant Therapeutical Approaches

As widely described previously, much evidence confirms the crucial role of ROS in almost every aspect of melanoma development and progression, acting both as a pro- and antitumorigenic factor. Therefore, idealistically, melanoma is a prototypical human cancer that could be managed from one side with pro-oxidant intervention to further increase the oxidative stress status to a level that is over the tolerance limit of tumoral cells, and on the other hand with antioxidants to counteract the increased ROS levels.

Exploring the literature, even only in the last decade, hundreds of molecules, bioactive compounds and phytochemicals targeting redox homeostasis have been tested for melanoma prevention and inhibition of disease progression. Concerning ROS-promoting therapies, several chemotherapeutic drugs can induce high levels of ROS, acting according to different mechanisms. A comprehensive review of all therapeutic approaches developed and tested for skin cancer treatment is outside of the scope of this work since most of them are already well-reported and were clearly discussed in different recent reviews [109,198,199]. Instead, in our contest, it is interesting to mention some redox-based therapies that do not involve the use of specific bioactive molecules, for instance, the ROS-promoting physical modalities, such as the well-known radiotherapy, photodynamic therapy (which combines a photosensitizer and light to generate ROS), hyperthermia, and gas plasma technology [200]. ROS produced by these therapeutical approaches induces an imbalance in redox equilibrium, promoting melanoma cell death.

#### 10.1.1. Radiotherapy

Regarding radiotherapy, melanoma exhibits radio-resistance, so it is not a valuable option except as an adjuvant or palliative approach [201] to relieve symptoms such as bone pain and brain dysfunction due to cancer metastases [202]. As it is known, radiotherapy induces the production of ROS in cancer cells, especially through the ionization of water molecules, resulting in DNA, lipid, and protein damage and ultimately leading to apoptosis [203]. Cancer cells equipped with a potent antioxidant cellular system can escape the damaging effects of radiation by scavenging ROS, leading to radio-resistance. In this context, the role of Nrf2 and its downstream enzymes in the radio-resistance of different human cancers has been documented [204,205]. Recently, it has been demonstrated that Nrf2 is also associated with radio-resistance in melanoma. The combination of Nrf2 knockdown and ionizing radiation treatment has a synergistic effect in reducing migration and invasion and in promoting apoptosis of B16-F10 murine melanoma cells [206]. Interestingly, radiotherapy may enhance the efficacy of immunomodulators since the produced ROS have a key role in fostering tumor-associated antigen release, antigen presentation and recognition, immune cell tumor infiltration and in avoiding immune suppression. As reported by Tagliaferri and colleagues [207], different clinical data are available in the literature, confirming that the combination of radiotherapy and immunotherapy seems to be a safe therapeutic option for malignant melanoma.

#### 10.1.2. Photodynamic Therapy

Photodynamic therapy (PT) has been investigated as an unconventional treatment for melanoma, characterized by limited side effects due to target action on cancerous tumor region. This treatment involves the administration of a photosensitizer light-sensitive drug to target cancer cells, combined with laser light used to excite photosensitizer, resulting in ROS production [208]. While PT has yielded good results in the treatment of over-exposed cancer (viz., head and neck cancers [209]), in the past, it was demonstrated to be less effective in the treatment of melanoma. The melanoma resistance to PT has been ascribed both to the presence of the melanin pigment that acts as a physical shield against PT and as ROS scavengers and to the presence of melanosomes that inhibit the photosensitizer accumulation [210]. Nevertheless, as reported by Baldea and colleagues [210], the discovery of improved photosensitizers (absorbing near-infrared (NIR) light to escape melanin protection) could encourage the use of this low-invasiveness method as a hopeful alternative treatment for melanoma. Interestingly, the use of a red PT light in combination with new photosensitizers was demonstrated efficient in several melanoma studies both in vitro and in vivo [210]. The efficacy of PT was also due to a specific aspect of its mechanism of action known as the “bystander effect” [211], which represents the ability of PT to induce damage not only in the target cells but also in adjacent cells through the diffusion of the ROS produced in the surrounding tissues. This characteristic clearly increases the efficacy of this treatment and decreases tumor recurrence. 

#### 10.1.3. Hyperthermia 

Hyperthermia is a local antitumoral treatment that uses exogenous heat sources that induce temperatures exceeding the physiological level, typically 40–43 °C, for nearly one hour. It can be considered a ROS-promoting therapy that increases the efficacy of other therapeutic approaches (viz., radiotherapy, chemotherapy, and immunotherapies) both in vitro and in vivo [212]. In late 1995, the multicenter randomized trial conducted on a total of 134 metastatic recurrent malignant melanoma lesions in 70 patients by Overgaard and colleagues demonstrated the combined effect of hyperthermia and radiotherapy in the treatment of melanoma; indeed, the authors observed an increase in the complete response rate from 35 to 62% and in the two-year local control rates from 28 to 46% [213].

#### 10.1.4. Gas Plasma Technology

Physical plasma is an excited gas state that may be produced by a constant supply of energy to the atoms or molecules of a neutral gas. This approach generates different ROS simultaneously that can induce local tumor oxidation [214]. A recent work demonstrated the efficacy of gas plasma technology in the treatment of melanoma both in vitro and in vivo [214]. In this study, five mitochondria-targeted drugs synergize with plasma treatment in B16F10 melanoma cells and in melanoma-bearing C57/Bl6 mice; in vitro, the combined therapy reduced cancer cell proliferation and promoted tumor toxicity and apoptosis compared with drug monotreatment. Oxidative distress was also observed, and irreversible oxidative damage confirmed the ROS-mediated antitumoral effect of this combined approach. The efficacy of the treatment was also confirmed by the histopathological analysis of cryosectioned tumor nodes of melanoma-bearing mice treated every day for a week with the combination of drug and gas plasma.

Although very promising, especially in combination all these ROS-promoting physical approaches still need more clinical studies to better define their efficacy in eliciting cell death selectively, reducing their possible side-effects.

#### 10.1.5. Nanotechnology Approaches

A new anti-melanoma therapeutic method based on redox manipulation that seems to offer novel and stimulating chances for the treatment of this human cancer is the nanotechnology approach [215]. Nanotechnology treatments could really be a good option in cancer therapy over the years since they can guarantee precise cancerous tissue targeting with minimal side effects. Moreover, due to their biological nature, nanomaterials can improve the bioavailability of specific compounds. Finally, the rapid release of drugs in tumorous tissues ensures an effective concentration for killing tumor cells. This is extremely important in the case of ROS-promoting therapies since it is possible to confine the increase in oxidative stress only to the tumor area without altering the redox state at systemic level.

One of the earliest studies on this novel therapeutical approach was by Wang and colleagues; they used cuprous oxide nanoparticles (CONPs) to treat mouse subcutaneous melanoma and metastatic lung tumors. After the intratumoral injection of CONPs, the authors observed a significant inhibition of the growth of the tumors and a higher survival rate in the mice. Moreover, since CONPs are rapidly cleared from the organs, they also perceived low hepatic and renal toxicities in mice. Regarding the molecular mechanism underlying CONPs, the authors demonstrated that they induced the reduction in melanoma progression by a mitochondrion-mediated apoptosis signaling pathway, causing Cyt-c release and activating caspase-3 and caspase-9. Moreover, by using copper sulfide nanocrystals, Wang and colleagues demonstrated that CONPs generated elevated ROS levels under NIR laser light irradiation, which recall the pathway for photodynamic therapy [216]. The advantage of these nanocrystals is that due to their very small size, they can penetrate deep into the tumor tissue, demonstrating a clear beneficial effect, especially for a tumor such as melanoma, which is characterized by a vertical growth in the deeper layers of the dermis. Even more attractive is the combination of nanotechnology plus the generating ROS cold atmospheric plasma (CAP) technique in the treatment of cutaneous melanoma. Recently, the synergism of CAP and silymarin nanoemulsion in inhibiting melanoma tumorigenesis was demonstrated. In vitro studies on human melanoma G-361 cells demonstrated that the co-treatment increased ROS production and upregulated the expression levels of pro-apoptotic proteins (i.e., caspases 3, 7, 8 and 9) while decreasing DNA damage. Moreover, the treatment diminished the protein expression levels of melanoma-specific biomarkers (e.g., BRAF) and the invasiveness of melanoma cells by overcoming the EMT. Also, in vivo co-treatment reduced tumor size, the expression levels of proliferation, and the specific biomarkers of this tumor [217]. For a detailed description of different ROS-generating nanotechnology systems, also combined with different physical methods, we refer to last year’s review by Pereira and colleagues [218].

### 10.2. The Antioxidant Therapeutical Approaches

As we already discussed in previous paragraphs, regarding the antioxidant therapeutical approaches, their use to treat melanoma is ambiguous, as some of the tested compounds have shown opposite effects depending on the stage of the cancer. Generally, it is accepted that the scavenger activity of antioxidants could prevent the initiation of melanoma, although this hypothesis is also far from being generally accepted. Indeed, while many in vitro studies corroborate this hypothesis, what emerges from different clinical trials failed to confirm the beneficial effects of antioxidants in preventing the occurrence of melanoma. The results of a systematic review that included published prospective cohort studies and randomized controlled trials concerning the role of antioxidant nutrients on the incidence of melanoma showed that there is no direct correlation between the intake of Vitamin C, Vitamin E, β-Carotene, Retinol, Vitamin A, Selenium, and melanoma risk in humans [219]. In addition, the VITamins And Lifestyle (VITAL) cohort study, which involved about 70,000 participants, demonstrated that there is no association between melanoma risk and dietary intake of vitamin A or carotenoids [220]. As clearly concluded by Hyeraci and colleagues [221], although many antioxidant compounds are used as supplements to reduce UV-induced photodamage (viz., erythema), to date, their effectiveness in preventing malignant melanoma has been shown in vitro, and only a few clinical trials confirm this assumption.

The till date usage (dietary or topical) of different antioxidants to counteract melanoma development and aggressivity has been largely described, and between them, the most outstanding example is N-acetyl cysteine (NAC). NAC is mostly known for its antioxidant and anti-inflammatory activity, which improves the maintenance of a cellular redox balance by restoring reduced GSH. In an ex vivo study, it has been revealed the potential role of NAC in protecting against pro-carcinogenic oxidative stress induced by UV exposure suggests that it could be considered an oral chemopreventive strategy for human melanoma [222]. A later in vivo study demonstrated that the daily injection of NAC in NSG mice subcutaneously transplanted with efficiently metastasizing melanoma cells derived from three patients without affecting tumoral growth promotes melanoma metastasis [132,223].

Another example of a well-researched double-faced antioxidant used in melanoma treatment is vitamin E. Preclinical studies suggested that vitamin E and its analogs could be of benefit for preventing melanoma development due to its strong photoprotective properties [224]. However, very recent data have demonstrated that Trolox, a Vitamin E analog, reduces ROS cellular levels and increases migration and invasion in human melanoma cell lines [225].

As reported by Cassidi and colleagues [226], selenium, a trace element with antioxidant properties and a key component of antioxidant enzymes, such as GPx and TRR, also plays a controversial role in melanoma development and progression. The authors observed in vivo a significant delay in the appearance of tumors in UV-irradiated mice upon topical application of selenium compared with animals treated with vehicle alone. This result suggests a preventive effect of selenium in the onset of melanoma; however, the presented data also suggested that selenium application may accelerate the growth of established tumors, proposing a harmful effect during melanoma progression.

Different papers have been published in the last decades focused on the characteristic redox arrangement of skin melanoma, and the role of pro-oxidant and antioxidant therapeutical approaches are also described [109,198,199,227,228]. What is emerging from all these works is that ROS can activate different oncogenic cellular pathways and, therefore, promote melanoma development, and the scavenger activity of antioxidants could suppress the initiation of this human cancer. An increasing amount of evidence confirms that a high ROS level supports all stages of melanoma development and progression. Indeed, melanocytes are particularly susceptible to oxidative stress due to the fundamental role of melanin synthesis and UV radiation in the generation of ROS. The continuous increase of oxidative stress that melanocytes are not able to counteract due to an unbalance between ROS generation and ROS detoxification exerts detrimental effects on normal cell functions and leads to tumor development. Therefore, in the early stage of melanoma, the addiction to antioxidants could rebalance redox homeostasis and avoid the rapid progression of this cancer. Meanwhile, transformed melanocytes are highly adaptive cells that may benefit from this boost of antioxidants to survive also in very stressful environments and to keep ROS cellular levels below a deadly threshold. Therefore, as melanoma grows, antioxidants switch from their physiological protective role to be detrimental substances supporting tumor progression and metastasis due to their intrinsic pro-survival characteristics. On the other hand, in this specific phase of melanoma progression, targeting the redox vulnerability of melanoma cells with specific approaches aimed at generating excessive ROS has emerged as a useful anti-cancer therapeutical strategy. Therefore, this interpretation is also far from completely persuasive; indeed, although pro-oxidant approaches have proven to be effective in inducing melanoma cell death, they can have important limits in clinical application due to the severe adverse effects on normal tissues and organs. Probably the most promising strategy should be focused on the delivery of anti-cancer drugs specifically to the target tumor tissue region to guarantee a balance between the cytotoxic and the adverse effects, raise effective benefits, and improve survival. Nevertheless, since the literature is divided on the role of antioxidant and pro-oxidant strategies in the treatment of melanoma, a more comprehensive understanding of the role of oxidative stress in this human cancer will guarantee the development of increasingly effective therapies. In conclusion, what we can summarize is that also, if apparently contradictory, the balanced redox homeostasis drives melanoma progression, while the unbalance between ROS production and ROS scavenger could mitigate the metastatic process of this human cancer.
